# The Isolation and Preparation of Samwinol from *Dracocephalum heterophyllum* and Prevention on Aβ_25–35_-Induced Neuroinflammation in PC-12 Cells

**DOI:** 10.3390/ijms231911572

**Published:** 2022-09-30

**Authors:** Chengzhao Li, Jun Dang, Yue Lv, Yan Fang, Chengjun Ma, Qilan Wang, Gang Li

**Affiliations:** 1 Center for Mitochondria and Healthy Aging, College of Life Sciences, Yantai University, Yantai 264005, China; 2 Qinghai Provincial Key Laboratory of Tibetan Medicine Research, Key Laboratory of Tibetan Medicine Research, Chinese Academy of Sciences, Northwest Institute of Plateau Biology, Xining 810001, China

**Keywords:** *D. heterophyllum*, PC-12 Cells, Aβ_25–35_, neuroinflammatory, oxidative stress

## Abstract

*Dracocephalum heterophyllum* (*D. heterophyllum*) is a traditional Chinese Tibetan medicine that has been used for the treatment of lymphitis, hepatitis, and bronchitis. However, only a few selected chemical components are currently obtained from *D. heterophyllum*, which limits its further pharmacological applications. In this study, we have obtained samwinol from *D. heterophyllum* by medium- and high-pressure liquid chromatography separation for the first time. Thereafter, we investigated the protective actions of samwinol against amyloid beta protein fragment 25–35 (Aβ_25–35_) induced neurotoxicity in cultured rat pheochromocytoma PC-12 cells and explored its underlying mechanisms of action. The results indicated that samwinol could increase cell viability and inhibit the production of reactive oxygen species (ROS) and mitochondria-derived ROS, as assessed by MTT assay, Giemsa staining, and flow cytometry assay. Through Western blot analysis, it was found that samwinol substantially inhibited the phosphorylation of ERK(1/2) and promoted the expression of HO-1 and Nrf2. The data obtained from molecular docking were also consistent with the above conclusions. All of these results showed that samwinol from *D. heterophyllum* can display significant anti-neuroinflammatory and antioxidant activities in vitro, which are associated with the suppression of ERK/AKT phosphorylation and the activation of the Nrf2/HO-1 signaling pathway. In the future, additional in-depth mechanism studies will be carried out to provide more evidence for the potential of samwinol in the treatment of Alzheimer’s disease.

## 1. Introduction

*Dracocephalum heterophyllum* (*D. heterophyllum*) is a traditional Tibetan medicine (TTM) called “Ao-Ga” or “Ji-Mei-Qing-Bao”, which is widely distributed in Xinjiang, Tibet, Qinghai, Gansu, and other provinces of China. It has been used in Tibetan medicine for the treatment of various diseases such as jaundice, liver disease, lymphangitis, mouth ulcers, and dental disease [1]. A number of prior reports have indicated that it also has antioxidant activity [2,3], anti-diabetic activity [4], inhibits hepatitis, and improves autoimmune diseases [5]. In addition, previous studies related to the analyses of phytochemical constituents have revealed that different flavonoids, alkaloids, triterpenes, and phenylpropanoids could be isolated from *D. heterophyllum*, but the chemical constituents currently obtained from *D. heterophyllum* are limited, which are not enough to support the subsequently reported pharmacological activities and pharmacodynamics mechanism research. Therefore, it is necessary to use a fast and efficient method to isolate pure compounds from *D. heterophyllum* to establish its potential in the management of various diseases.

The high-performance liquid chromatography-1,1-diphenyl-2-picrylhydrazine (HPLC-DPPH) screening system enables rapid analytical evaluation of target-specific potential antioxidants [6]. In this method, the ability of the compound to scavenge free radicals is measured by the decrease in absorbance at 517 nm after the addition of 1,1-diphenyl-2-picrylhydrazine (DPPH) to the HPLC pathway. This method has been widely used to detect the presence of various antioxidants in many plants and foods, and it has screened several new active compounds with antioxidant activity [7,8]. In addition, one of the best chromatographic methods currently available for the extraction of single compounds from complex samples is preparative HPLC, which has the advantages of good separation, real-time monitoring, and high reproducibility [9]. This can also be used for the separation and purification of various chemical components of *D. heterophyllum*.

Alzheimer’s disease (AD) is a prevalent chronic neurological condition linked to a progressive decrease in cognition and memory [10]. Progressive memory loss, poor executive function, and difficulties performing daily activities are clinical symptoms of AD, and early signals of AD development include changes in thinking or unconscious behavior, memory deficiencies for new knowledge, as well as dysfunctional alterations in both language and speech [11]. The main pathological characteristics associated with AD include Aβ plaques, neurogenic fiber tangles, and neuronal loss [12], with cerebrovascular amyloidosis, inflammation [13], and synaptic lesions of nerves [14]. There are several hypotheses proposed to explain the cause of AD, including the cholinergic hypothesis, the β-amyloid hypothesis, and the Tau protein hypothesis [15]. According to the β-amyloid hypothesis, the main component of amyloid beta (Aβ) is senile plaques, which are primarily composed of 36–43 amino acid residues [16]. A large body of medical evidence suggests that Aβ can play a significant role in the pathogenesis of AD [17,18,19].

Microglia are the only resident macrophage-type cells present in the central nervous system. Activated microglia can take up soluble or fibrillar Aβ through the process of phagocytosis and dissolve these peptides through the proteasomal pathway [20]. Aβ induces significant neurodegeneration by activating microglia, which trigger neurotoxicity through the release of inflammatory mediators such as different cytokines and reactive oxygen species (ROS) to promote the development of AD [21]. A growing number of investigations have described various pharmacological strategies to alleviate AD by treating neuroinflammation and oxidative stress [22,23,24].

To the best of our knowledge, there have been no prior reports describing the pharmacological effects of various components from *D. heterophyllum* in AD. The aim of this work was to extract the different monomeric compounds with antioxidant activity from *D*. *Heterophyllum* and to evaluate their possible effects on neuroinflammation by using PC12 cells.

## 2. Results

### 2.1. Sample Pretreatment with Medium-Pressure Liquid Chromatography

**Figure 1:**
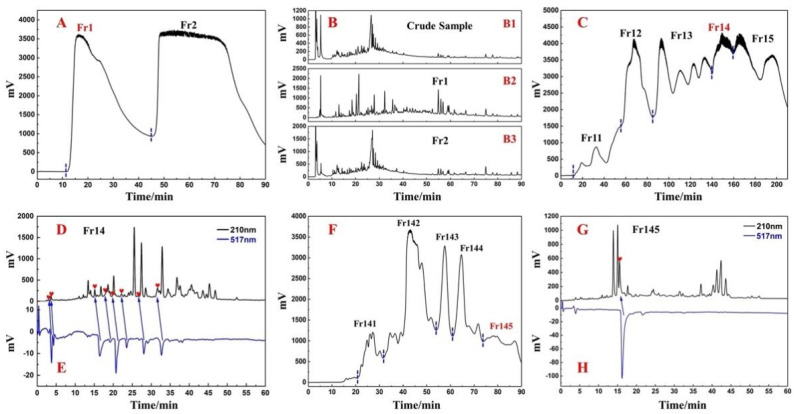
*D. heterophyllum* extract separation chromatogram (**A**) with silica gel medium-pressure liquid chromatography. Analytical chromatograms (**B**) of the crude samples, Fr1 and Fr2, respectively. The pretreatment chromatogram (**C**) of Fr1 with MCI GEL^®^CHP20P middle-pressure liquid chromatography. The analytical chromatogram (**D**) on the ReproSil-Pur C18 AQ analytical column and DPPH radical scavenging profile (**E**) of fraction Fr14 sample. The pretreatment chromatogram (**F**) of Fr14 with diol middle-pressure liquid chromatography. The analytical chromatogram (**G**) on the ReproSil-Pur C18 AQ analytical column and DPPH radical scavenging profile (**H**) of fraction Fr145 sample. The conditions used for HPLC1 (**D**,**G**): mobile phase A: HPLC-grade water, B: ACN; gradient: 0–60 min, 40–75% B; monitoring wavelength: 210 nm; flow rate: 1.0 mL/min; column temperature: 30 °C Conditions for HPLC2 (**E**,**H**): the monitoring wavelength: 517 nm; DPPH solution flow rate: 0.8 mL/min.

Considering the environment-friendly characteristics of methanol, it was selected as a solvent to extract the whole plant. Finally, a crude sample of about 1.3 kg was obtained from 10.0 kg of dried *D. heterophyllum* whole plant at about a 13.4% extraction rate. Thereafter, the crude sample was subjected to the first pretreatment by medium-pressure liquid chromatography on the silica gel. The main purpose was to remove the various polymers and sugars, which are not our target substances. The removal of these components can simplify the subsequent separation steps. At the same time, visual separation was achieved on the preparative liquid chromatography, and the crude sample was separated into two fractions, Fr1 and Fr2, as shown in Figure 1A. Baseline separation can be achieved between the two fractions. Finally, all the crude samples were processed, and two different fractions were obtained, of which the target fraction Fr1 weighed 205.5 g (15.8% recovery). On the contrary, the separated Fr1, Fr2, and crude samples were analyzed using the same conditions, and the analytical conditions were gradient elution on a ReproSil-Pur C18 AQ analytical column. The analytical chromatograms are shown in Figure 1B. Among them, Figure 1(B1) demonstrates the analytical chromatogram of the crude sample. The composition of the crude sample was complex, but Figure 1(B2,B3) shows the analytical chromatograms of Fr1 and Fr2. Although the compositions of the two fractions were found to overlap slightly, the separation of components in the crude sample was largely achieved.

The Fr1 at this stage still contained a large amount of chlorophyll. Hence, if Fr1 was prepared directly in the next step, it was possible that chlorophyll might adsorb on the stationary phase of the preparation column and cause damage to the preparation column, so it was necessary to remove the chlorophyll first. The MCI GEL^®^ CHP20P column was selected for the second pretreatment of Fr1 to remove the chlorophyll, whereas Fr1 was segmented to refine the fractions. Figure 1C shows a separation chromatogram. Five fractions (Fr11, Fr12, Fr13, Fr14, and Fr15) were collected after nine repetitions of the MCI GEL^®^ CHP20P pretreatment. Among them, Fr14 was 44.2 g, which was of high quality. We selected Fr14 as the target fraction for the subsequent separation.

**Figure 2:**
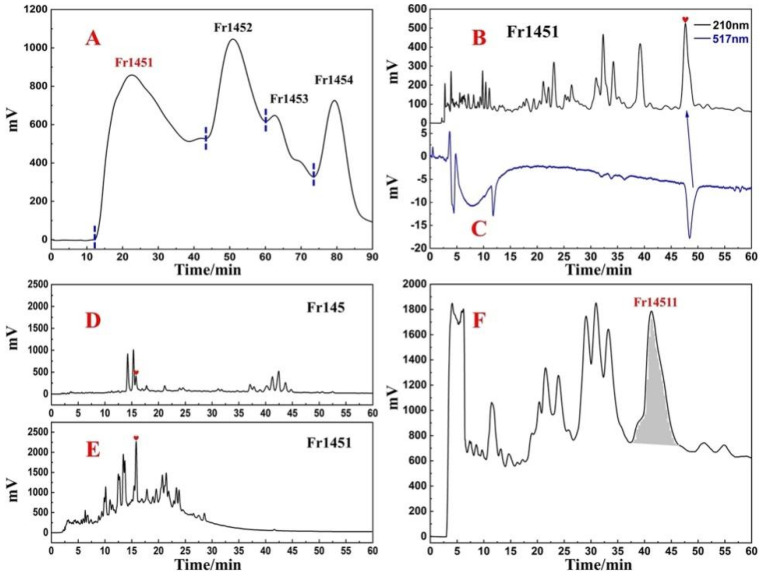
The preparative chromatogram (**A**) of fraction Fr145 sample on the Spherical C18 column. The analytical chromatogram (**B**) on the ReproSil-Pur C18 AQ analytical column and 1,1-diphenyl-2-picrylhydrazyl (DPPH) radical scavenging profile (**C**) of fraction Fr1451 sample. The conditions used for HPLC1 were as follows (**B**): mobile phase A: HPLC-grade water, B: ACN; gradient: 0–60 min, 40% B; monitoring wavelength: 210 nm; flow rate: 1.0 mL/min; column temperature: 30 °C. Conditions for HPLC2 (**C**): monitoring wavelength: 517 nm; DPPH solution flow rate: 0.8 mL/min. Analysis and comparison of chromatograms of Fr145 (**D**) and Fr1451 (**E**). The preparative chromatogram (**F**) of fraction Fr1451 sample on the ReproSil-Pur C18 AQ analytical column.

In order to analyze the Fr14, 100 mg of Fr14 was solubilized in 0.1 mL MeOH and filtered through a 0.45 mm filter. Thereafter it was analyzed on an online HPLC-DPPH system using a ReproSil-Pur C18 AQ analytical column under optimal chromatographic conditions. The results have been shown in Figure 1D,E, and Fr14 had several negative peaks at 517 nm, thus indicating that there were several antioxidant peaks in Fr14. In addition, it was observed that the composition of Fr14 was rather complex, and it was difficult to directly separate the antioxidant peak. This requires the next step of pretreatment of Fr14 to enrich the various active components. As shown in Figure 1F, the diol medium-pressure chromatographic column divided Fr14 into five sub-fractions, and Fr145 (4.1 g) was selected as the next target fraction. The antioxidant peak of Fr145 was also recognized online on the HPLC-DPPH system using a ReproSil-Pur C18 analytical column AQ. Figure 1G,H showed a negative peak at 517 nm at around 17 min, thereby indicating that Fr145 contained an antioxidant peak (antioxidant component). Next, a Spherical C18 medium-pressure column was used for the next step of enrichment of the various active ingredients. As shown in Figure 2A, Fr145 was divided into four sub-fractions. After analysis using the online HPLC-DPPH system, it was found that the active components identified in Fr145 were enriched in Fr1451. The screening results have been shown in Figure 2B,C; there was a strong negative peak at 517nm during 45-50 min in Fr1451. To verify the reliability of these results, Fr145 and Fr1451 were compared under similar chromatographic conditions as in Figure 1G. The analysis results have been shown in Figure 2D,E. The activity peak at 17 min in Fr145 was indeed present in Fr1451. This provided direction for the subsequent separation and purification of active chromatographic peaks.

### 2.2. Target Preparation of Samwinol of Fr1451 with High-Pressure Liquid Chromatography

The analytical conditions in Figure 2B can effectively separate the active peaks from the impure peaks that exist next to them. We performed the preparation of Fr1451 on a ReproSil-Pur C18 AQ preparative column with linear amplification of the above conditions, and Figure 2F depicted the preparative chromatogram of the Fr142 fraction. The retention times of the active peaks observed on the ReproSil-Pur C18 AQ preparative column (Figure 2F) and the ReproSil-Pur C18 AQ analytical column (Figure 2B) were found to be similar in comparison to Figure 2B. After preparation, 16.1 mg of Fr14511 was finally obtained with a recovery of 4.3%.

**Figure 3:**
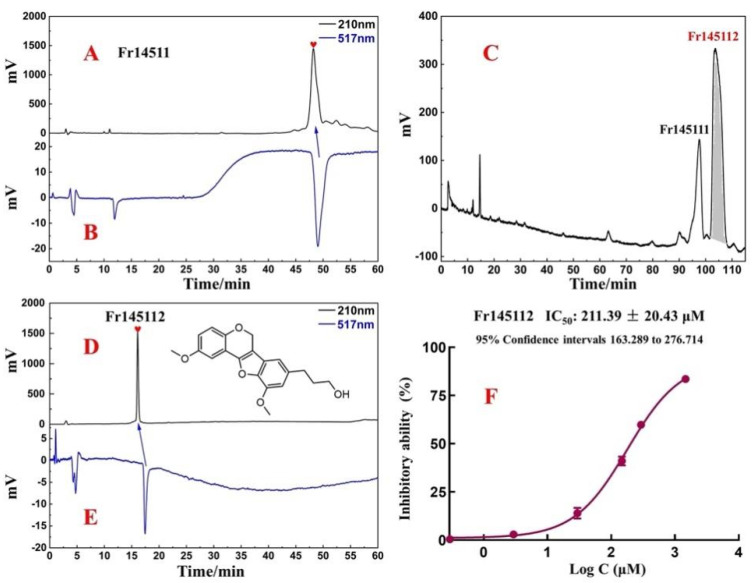
The analytical chromatogram (**A**) on the ReproSil-Pur C18 AQ analytical column and 1,1-diphenyl-2-picrylhydrazyl (DPPH) radical scavenging profile (**B**) of the fraction Fr14511 sample. The preparative chromatogram (**C**) of fraction Fr14511 sample on the ReproSil-Pur C18 AQ analytical column. Purity and DPPH inhibitory activity verification chromatogram of the isolated Samwinol (**D,E**) on ReproSil-Pur C18 AQ analytical column. The conditions used for HPLC1 were as follows (**A**,**D**): mobile phase A: HPLC-grade water, B: ACN; gradient: 0–60 min, 40–80% B; monitoring wavelength: 210 nm; flow rate: 1.0 mL/min; column temperature: 30 °C. Conditions for HPLC2 (**B**,**E**): monitoring wavelength: 517 nm; DPPH solution flow rate: 0.8 mL/min. DPPH inhibitory activities and 50% inhibition (IC_50_) values of Fr145112 (**F**) at the different concentrations (μM).

Next, both the purity analysis and activity verification of Fr14511 were carried out using an online HPLC-DPPH system. As shown in Figure 3A,B, there was a strong negative activity peak at 517 nm. However, from Figure 3A, this chromatographic peak was found to be impure. It appears that the two chromatographic peaks were merged, and there were some small magazine peaks behind the main peak, so Fr14511 needs to be further purified.

The chromatographic conditions were further optimized on the ReproSil-Pur C18 AQ column. When 40% acetonitrile was used for isocratic elution, two distinct chromatographic peaks were successfully separated in the range of 90–110 min (Figure 3C). After the linear amplification of the chromatographic conditions, Fr14511 was purified. The main peak was Fr145112, the final collected fraction solution was concentrated and dried to obtain 3.74 mg of Fr145112, and the recovery rate was 23.2%.

### 2.3. Purity, Activity, and Structure of Fr145112

Isolated Fr145112 was re-evaluated for purity and activity using the online HPLC-DPPH system with the ReproSil-Pur C18 AQ analytical column. It was observed to possess purities greater than 95%, as illustrated in Figure 3D,E. To elucidate the structure of the target compound, Fr145112 were identified by comparing their ESI-MS and NMR spectrum data with the previously reported data. The supporting information includes the full spectra generated in this study. Fr145112 had NMR and MS data that matched with the data for samwinol. The chemical structure is shown in Figure 3D.

Fr145112: (samwinol, 3.74 mg, yellow powder, ESI-MS *m*/*z*: 293.13 [M + Na]^+^). 1H NMR (600 MHz, DMSO-*d*_6_) δ: 7.36 (1H, d, *J* = 1.6 Hz, 2-H), 7.24 (1H, dd, *J* = 8.1, 1.6 Hz, 4-H), 7.07 (1H, brs, 6′-H), 6.93 (1H, d, *J* = 8.1 Hz, 5-H), 6.77 (1H, brs, 2′-H), 4.63 (2H, s, 9-H), 3.95 (3H, s, 3′-OCH3), 3.86 (2H, m, 9′-H), 3.85 (3H, m, 3-OCH3), 2.70 (2H, t, *J* = 7.6 Hz, 7′-H), 2.70 (2H, m, 8′-H). 13C NMR (151 MHz, DMSO-*d*_6_) δ: 154.1 (C-7), 147.8 (C-3), 147.7 (C-6), 144.3 (C-3′), 140.4 (C-4′), 138.1 (C-1′), 131.3 (C-5′), 121.0 (C-1), 120.1 (C-4), 115.9 (C-5), 111.2 (C-8), 110.8 (C-6′), 110.6 (C-2), 107.6 (C-2′), 60.2 (C-9′), 55.7 (-OCH3), 55.5 (-OCH3), 55.5 (C-9), 34.9 (C-8′), 32.1 (C-7′). These data were consistent with the previously reported data for samwinol [25].

To further evaluate the DPPH scavenging activity of samwinol, the protocol was performed by using a slight modification [26,27,28]. As shown in Figure 3F, samwinol exhibited significant antioxidant activity, and the IC_50_ value was 211 ± 20.43 µM. The presence of the hydroxyl groups on the branched chains might have enhanced the antioxidant activity of the analyzed compounds. Both chromatographic and chemical methods have confirmed that the compound has good antioxidant activity, and substantial antioxidant activity could be the basis for the treatment of several other diseases. Using the existing compounds, other pharmacological activities can be further explored.

### 2.4. Binding Ability of Samwinol to Antioxidant Proteins

**Figure 4:**
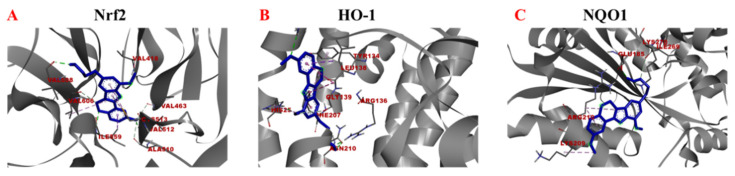
Molecular docking analysis of Samwinol binding to Nrf2 (**A**), HO-1 (**B**), and NQO1 (**C**), respectively.

Since the above-ground part of D. heterophyllumand has been reported to treat lymphadenitis, hepatitis, and bronchitis, it was speculated that samwinol might also possess substantial antioxidant effects. Molecular docking analyses were performed to evaluate the potential interaction between samwinol and antioxidative protein Nrf2, HO-1, and NQO 1. The protein conformations of Nrf2, HO-1, and NQO1 and the possible amino acid sites of these antioxidant proteins bound to samwinol are depicted in Figure 4. The binding energy and ligand interaction have been shown in Table 1. Samwinol possessed more potential binding sites for Nrf2 and HO-1 and lowered the binding energy more than NQO1, which suggested that samwinol might influence the antioxidant pathway from Nrf2 to HO-1.

### 2.5. The Effects of Samwinol on PC12 Cells

**Figure 5:**
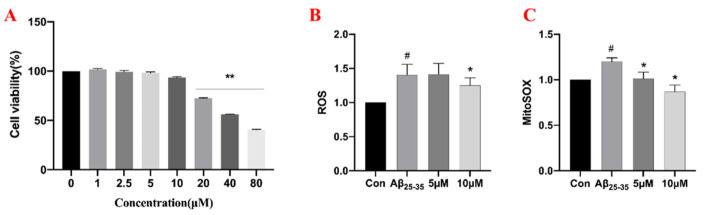
Effect of samwinol on cell viability of PC12 cells as measured by MTT assay (**A**). ** *p* < 0.01, vs. normal group. The impact of samwinol on production of ROS (**B**) and Mito-ROS (**C**) in PC12 cells. # *p* < 0.05, Aβ_25–35_ group vs. Con group (Control group); * *p* < 0.05, samwinol groups vs. Aβ_25–35_ group.

To detect the potential effects of samwinol on the viability of PC12 cells, an MTT assay was carried out with various concentrations of samwinol. The results in Figure 5A showed that the cell viability exhibited no significant changes when the concentration was less than 10 μM. Thus, 5 μM and 10 μM of samwinol were selected in the following experiments.

**Figure 6:**
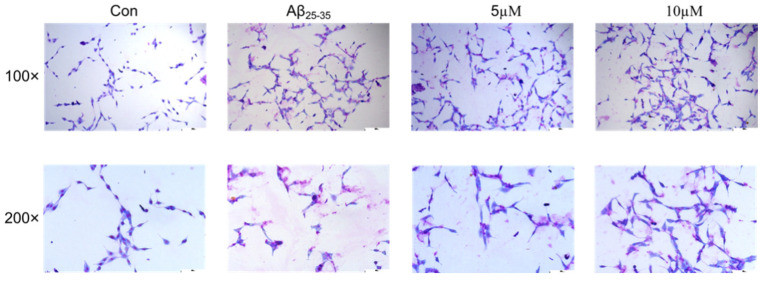
Detection of morphological changes in PC12 cells by Giemsa staining.

Aβ_25–35_ has been commonly used to build the AD model. The PC12 cells were stained with Giemsa staining solution to observe the possible morphological changes. The results in Figure 6 showed that cell death, leakage of material from the nucleus, shortening of cell synapses, and cell crumpling were observed in the Aβ_25–35_ group. After the samwinol treatment, the cell density was found to increase, cell synapses were restored, and the cells gradually became round in appearance following the treatment.

The mitochondrial respiratory chain is one of the major sources of cellular ROS, which consists of a group of unstable molecules, including hydrogen peroxide (H_2_O_2_), hydroxyl radicals (OH^−^), singlet oxygen (O^2^), and superoxide (O^2−^) [29]. Under normal physiological conditions, the balance between ROS production and ROS clearance is tightly controlled [30]. When the body is in a state of oxidative stress, high levels of ROS can lead to oxidative damage. The production of ROS is central to the progression of several inflammatory diseases. ROS produced by cells is involved in the host defense response [31]. Therefore, the changes in ROS can be used to demonstrate the anti-inflammation ability of samwinol.

To verify the previous speculation, the ROS and Mito-ROS levels in PC12 were detected by flow cytometry. The results in Figure 5 show that the trend of the production of ROS (B) and Mito-ROS (C) results were consistent. The production of ROS and Mito-ROS in the Aβ_25–35_ group were obviously higher than that of the control group, and 10 μM of samwinol could obviously reverse the situation. These findings suggested that Aβ_25–35_ causes an increase in ROS production in PC12 cells, which was primarily derived from mitochondria, and the treatment of samwinol can effectively alleviate oxidative stress.

### 2.6. Western Blot Assay for Protein Expression in PC12 Cells

Mitogen-activated protein kinase (MAPKs) are a family of serine/threonine protein kinases, which include members of the extracellular receptor-activated kinase (ERK) 1/2, p38, and c-Jun N-terminal kinase (JNK) [32]. It is widely believed that MAPKs can play critical roles in the regulation of inflammation and induce the expression of various inflammatory mediators and pro-inflammatory cytokines [33]. The experimental results showed that the expression of p-ERK (1/2) was upregulated in the model group compared with the control group, and 10 μM of samwinol demonstrated superior anti-inflammatory activity than that of 5 μM. Samwinol might exert anti-inflammatory effects by inhibiting the activation of p-ERK (1/2). In addition, samwinol also suppressed the activation of p-AKT induced by Aβ_25–35_ in a concentration-dependent manner.

**Figure 7:**
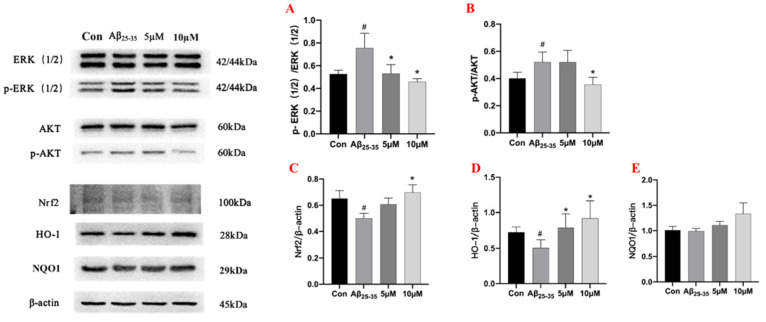
Effects of samwinol on the expression of proteins in PC12 cells. Semi-quantitative analysis of *p*-ERK/ERK (**A**), *p*-AKT/AKT (**B**), Nrf2 (**C**), HO-1(**D**), and NQO1(**E**). # *p* < 0.05, Aβ_25–35_ group vs. Con group (Control group); * *p* < 0.05, samwinol treatment group vs. Aβ_25–35_ group.

In mammals, nuclear-associated factor 2 (Nrf2) plays a pivotal role in counteracting oxidative stress mechanisms, and Nrf2 is a defense system used to maintain cellular redox homeostasis [34]. During this process, oxidative stress can activate Nrf2 gene expression and transcriptional activity in the body. Next, Nrf2 can regulate the expression of antioxidant proteins such as heme oxygenase 1 (HO-1), and NQO1 can be activated in response to ROS to protect the cells from inflammation-induced oxidative stress [35]. The results in Figure 7 showed that samwinol markedly increased the expression of Nrf2 and HO-1. Since Aβ_25–35_ caused oxidative stress in the cells, it led to the inhibition of Nrf2 expression. However, samwinol reversed this change, which could augment the expression of Nrf2 and promote the translocation of Nrf2 into the nucleus. Nrf2 can then increase the expression of antioxidant proteins HO-1 to counteract oxidative stress in PC12 cells. The protein expression of NQO1 displayed no significant difference (*p* > 0.05) between the Aβ_25–35_ group and the samwinol-treated group. Thus, these results showed that samwinol exhibited minimal effects on the expression of NQO1 protein.

## 3. Discussion

In this study, a quick and efficient approach of the medium- and high-pressure liquid chromatography combined with an online HPLC–DPPH system was used to recognize, separate, and purify antioxidative lignan from *D. heterophyllum*. The isolated compound was identified as samwinol. Samwinol is a lignin that was originally isolated from *Sambucus williamsii* [25]. Natural polyphenols can serve as potent antioxidants with the advantages of being non-toxic, highly safe, naturally available, and found in abundance in nature. The phenolic hydroxyl groups of lignin can contribute to its antioxidant activity [36]. The above-ground parts of *Dracocephalum heterophyllum* have been reported to be used for the treatment of hypertension, lymphadenitis, hepatitis, and bronchitis in Tibetan medicine [37]. The antioxidant capacity of samwinol isolated from *D. heterophyllum* was also established by molecular docking and DPPH assays for the first time.

According to the existing literature reports, we can see that Aβ_25–35_ can trigger an inflammatory response in microglia, leading to the phosphorylation of the MAPK family (ERK (1/2)) [38]. The inflammatory response will cause a significant upregulation of ROS and Mito-ROS along with intracellular oxidative stress [39] ROS can act as a potential signaling molecule to inhibit the expression and nucleation of Nrf2 protein and the expression of the antioxidant protein HO-1 protein downstream of Nrf2 [40].

In our study, the activity of the compounds was initially corroborated using the compounds obtained by isolation for the first time around the antioxidant system in the inflammatory process of AD. However, the development process of AD is complicated, and oxidative stress is also only one aspect of the interaction. This study only used the PC12 cells model to conduct preliminary activity evaluation and study in vitro. Combined with the molecular docking antioxidant, the results were in good agreement with the cell experiment, suggesting that our compound may have better findings in antioxidants. More studies are needed in the future to reveal the detailed mechanism of action of samwinol in the treatment of AD.

## 4. Materials and Methods

### 4.1. Instrumentation and Reagents

The preparative liquid chromatography used in this study included two NP7000 prep-HPLC pumps, an NU3000 ultraviolet-visible (UV-Vis) detector, and an LC workstation (Hanbon Science & Technology Co., Huaian, China). The online HPLC-DPPH system was constructed with Essentia LC-16 (Shimadzu Instruments, Co., Shanghai, China) and LC-10AD HPLC equipment (Shimadzu Instruments, Co., Kyoto, Japan). The device was equipped with two binary gradient pumps, a UV-Vis detector, a column oven, and an LC workstation. The two HPLCs were connected by a triple valve and a polyether ether ketone reaction coil (18.0 m × 0.25 mm i.d.). LC-16 was used for HPLC analysis, and LC-10AD was used to obtain DPPH screening chromatograms. ESI-MS analysis was performed by a Waters QDa Electrospray Ionization Mass Spectrometer (ESI-MS) (Waters Instruments Co., Milford, MA, USA). The 1H and 13C NMR spectra were acquired on a 600 MHz Bruker Avance spectrometer (Bruker Instruments Co., Berlin, Germany). The solvent for NMR was DMSO-*d*_6_. UV absorbance values were obtained with a Readmax 1900 microplate reader (Flash, Co., Shanghai, China).

The diol (50 × 500 mm, 25 μm) column was obtained from the ACCHROM Corporation (Beijing, China). Silica gel (100–200 mesh) material was purchased from the Qingdao Ocean Chemical Company (Qingdao, China). MCI GEL^®^ CHP20P (120 μm) separation material was obtained from the Mitsubishi Chemical Corporation (Tokyo, Japan). Two ReproSil-Pur C18 AQ columns (4.6 × 250 mm, 5 μm, and 20 × 250 mm, 5 μm) were purchased from the Maisch Corporation (Berlin,Germany). A spherical C18 column (50 × 500 mm, 50 μm) was obtained from SiliCycle (Quebec, QC, Canada).

DPPH was purchased from Sigma-Aldrich (Steinheim, Germany). Analytical and HPLC grade ethanol (MeOH), analytical grade dichloromethane (CH_2_Cl_2_), analytical grade, and HPLC grade ACN, analytical grade n-hexane, and analytical grade ethyl acetate were purchased from the Kelon Chemical Reagent Factory (Chengdu, China). HPLC grade H_2_O was prepared using a water purifier from Moore (Chongqing, China).

### 4.2. Plant Sample Preparation and Medium Pressure Liquid Chromatography Pretreatment

Our group has previously successfully completed the isolation and identification of several new active compounds [7,8]. Based on the previous studies, the separation method of *D. heterophyllum* was adjusted to some extent in this study. The herb of *D. heterophyllum* was collected from North Mountain in Huzhu, Qinghai, and verified by Professor Lijuan Mei of Northwest Plateau Institute of Biology. The sample (nwipb-2016-10-10) was kept in the Qinghai–Tibet Plateau Biological Museum. The collected whole herbs were dried in the shade, weighed, and thereafter all the whole herbs (10.0 kg) were crushed and extracted three times with 100% methanol, each using 80.0 L of solvent, and the extraction time was 12 h. Finally, 240.0 L of extract solution was collected, filtered, and concentrated at 40 °C using a rotary evaporator. When the volume of the concentrate was reduced to 5.0 L, 1.5 kg of amorphous silica gel was added, mixed well, and dried in a 40 °C oven to obtain 2.8 kg of silica gel sample mixture. The silica gel mixture was pretreated by silica medium-pressure liquid chromatography using MeOH and CH_2_Cl_2_ as mobile phases, and the elution conditions were 0–30 min, 0% methanol; 30–60 min, 0–100% methanol; and 60–90 min, 100% methanol, flow rate kept at 57.0 mL/min; the single sample loading was 65 g, and the chromatogram was recorded at 210 nm. After repeating this process 43 times, fraction Fr1 (205.5 g) was obtained as the subsequent separation material.

Fraction Fr1 (205.5 g) was dissolved in 2.0 L MeOH. Then 234.0 g of amorphous silica gel was added to the methanol solution of Fr1, mixed, and dried in an oven at 40 °C to finally obtain 439.5 g of a dry mixture. The mixture was further pretreated using MCI GEL^®^ CHP20P medium-pressure columns (49 × 460 mm). The mobile phase used was MeOH/H_2_O with elution conditions of 0–150 min, 20–100% methanol, and 150–210 min, 100% methanol. The flow rate and wavelength were the same as in the first step of pretreatment, and the single loading was 55.0 g. After repeating the process eight times, fractions Fr11-Fr15 were obtained and concentrated by drying. The fraction Fr14 (44.2 g) was selected for the subsequent isolation. Similarly, fraction Fr14 (44.2 g) was dissolved in 400.0 mL of MeOH, mixed with 74.0 g of amorphous silica gel, and dried in an oven at 40 °C. The dry mixture (118.2 g) was subjected to a third pretreatment with a diol medium-pressure column (50 × 500 mm) using N-hexane/ethyl acetate as a mobile phase. The linear gradient elution conditions were 0–90 min and 0–100% ethyl acetate. The flow rate was 57.0 mL/min, and the chromatogram was recorded at 280 nm. After the two replicate separations, the target fractions (Fr145) were collected, pooled, and concentrated to yield 4.1 g of enriched sample with 9.3% recovery.

Fr145 was further separated using the Spherical C18 medium-pressure column (50 × 500 mm) preparative column. HPLC grade water and ACN were used as mobile phases A and B, respectively. The gradient elution step for Fr145 was 0–90 min, 50–80% B. The equilibration time was 15 min at a flow rate of 57.0 mL/min. The chromatogram was recorded at 210 nm. Thereafter, 374 mg of Fr1451 was obtained.

### 4.3. High-Pressure Liquid Chromatography Separation and Purification of Samwinol from Fr1451

Fr1451 were separated by using a ReproSil-Pur C18 AQ (20 × 250 mm, 5 μm) preparative column. Mobile phase A was HPLC grade water, and mobile phase B was ACN. The isocratic elution step was performed by using 40% B for 60 min. The flow rate of the eluent was maintained at 19.0 mL/min, and the elution process was measured at 210 nm. Finally, 16 mg of Fr14511 was obtained. Then, Fr14511 (16 mg) was completely dissolved in MeOH (1.0 mL), eluted isocratically with 30% ACN/H_2_O on the ReproSil-Pur C18 AQ (20 × 250 mm, 5 μm) preparative column for 120 min, the chromatogram was recorded at 210 nm, and the second main peak (Samwinol) was collected and dried to obtain 3.45 mg of the compound.

### 4.4. Assessment of Purity and Activity of Samwinol

The purity and activity of samwinol were assessed by using an online HPLC-DPPH system. The analysis was performed by using a ReproSil-Pur C18 AQ (4.6 × 250 mm, 5 μm) column. The mobile phase system was ACN/H_2_O, with a linear gradient based on 40–80% ACN, an elution time 60 min, and a flow rate 1.0 mL/min. The absorbance was measured at 210 nm. The concentration of DPPH was 25 μg/mL, and the eluate flowed at a rate of 0.8 mL/min. DPPH ethanol solution chromatogram was recorded at 517 nm.

DPPH was prepared in an ethanol solution at a concentration of 25 μg/mL, and the isolated Samwinol was prepared in solutions with different concentration gradients (0.1, 1, 10, 50, 100, and 500 μg/mL). The sample solution (30 μL) and the ethanol solution (70 μL) were added to the 96-well plate, respectively. Each sample was repeated three times. The mixed solution was then incubated in the dark for 30 min. Finally, the UV absorption value of the mixture was recorded at 517 nm, denoted as A. This experiment was repeated three times. The scavenging rate of the DPPH radicals has been represented by the following formula.

(1) $$\mathrm{DPPH}\;\mathrm{inhibition}\;(\%)=1-\left(\frac{A-A_{0}}{A_{1}}\right)\times 100\%$$

where A_0_ denotes the absorbance of the blank group (ethanol), A_1_ is the absorbance of the control group, and A is the absorbance of the sample solution.

### 4.5. Predicting the Binding Capacity of Samwinol to Antioxidant Proteins by Using Molecular Docking

The binding ability of samwinol and antioxidant proteins Nrf2, HO-1, and NQO1 was predicted by using a computerized molecular docking analysis, which was used to predict the antioxidant capacity of Samwinol. The ID of Nrf2 (ID: 4IQK), HO-1 (ID: 1N3U), and NQO1 (ID:2FUG) can be found in the protein data bank (https://www.rcsb.org/, accessed on 23 August 2022). First, irons and water were eliminated from the receptor. Next, the Kollaman charges and polar hydrogen were added. The Lamarckian genetic algorithm, the pseudo-Solis, and Wets methods were used for the minimization, setting the parameters to default. According to the dock score values, a total of 100 peptide conformations were obtained. Model development predefined the minimum binding energy. These results were visualized and analyzed using Discovery Studio 2020.

### 4.6. Cellular Antioxidant Activity Assays

#### 4.6.1. Culture of PC12 Cells and Aβ_25–35_ Induced AD Model

Aβ_25–35_ (A107852, Aladdin, Shanghai, China) was dissolved in the ultrapure water used for reserve fluid (1 mM). The reserve fluid was stored at −20 °C and incubated at 37 °C for 72 h before use. The PC-12 cell line was purchased from the Cell Bank of the Chinese Academy of Science. The cells were maintained at 37 °C in a 5% CO_2_ incubator with saturated humidity. The culture medium was DMEM (12800-082, Gibco, Beijing, China) with 1% penicillin and 10% fetal bovine serum.

#### 4.6.2. Effects of Samwinol on Cell Viability

The PC12 cells were seeded in 96-well plates at a density of 5 × 10^3^cells/well and then incubated with samwinol at various concentrations (1–80 μM) for 24 h, respectively. After incubating, 10 μL of MTT solution (5 mg/mL) was added to each well for 4 h, and the medium was aspirated and shaken with 100 μL DMSO (67-68-5, Sinopharm, Beijing, China) for 15 min, and the absorbance value was measured at 490 nm using a microplate reader (Molecular Divice, Sunnyvalr, CA, USA).

#### 4.6.3. Morphological Changes as Analyzed by GIEMSA Staining

The PC12 cells were inoculated in 24-well plates (5 × 10^4^ cells/well), samwinol was incubated in advance for 2 h, then Aβ_25–35_ was added and incubated for 24 h. The plates were washed twice with PBS when the medium was aspirated, and 4% paraformaldehyde (PH0427, Phygene, Fuzhou, China) was added to fix at 4 °C for one h. Giemsa staining solution was added for 10 min and washed with PBS. Finally, the cell morphology was observed under the microscope (Leica Microsystems CMS GmbH, Wetzlar, Germany).

#### 4.6.4. Measurement of ROS and Mito-ROS Levels by Flow Cytometry

The PC12 cells were inoculated in 6-well plates (1 × 10^5^ cells/well), samwinol was incubated in advance for 2 h, then Aβ_25–35_ was added and incubated for 2 h. The cells were then incubated with a DCFH-DA probe (5 μM) and Mito-sox probe (10 μM) for 15 min and 30 min, respectively. Thereafter, the cells were digested for 5 min and collected for centrifugation. The supernatant was aspirated, and the cells were mixed well by adding PBS. ROS and Mito-ROS levels were assayed by flow cytometry (ACEA Biosciences, San Diego, CA, USA).

#### 4.6.5. Detection of Protein Expression by Western Blot Assay

Western blot was used to analyze changes in cellular signaling. Briefly, the total proteins were extracted by lysing the cells with cell lysis solution (P0013, Beyotime, Shanghai, China) with 1%PMSF (ST506-2, Beyotime, Shanghai, China), and the protein concentration was measured by the BCA Protein Assay Kit. (P0009, Beyotime, Shanghai, China). Then, 10% SDS-PAGE was used to separate the protein samples and transfer the target protein to the PVDF membrane (ISEQ00010, Millipore, Ireland) under a 250 mA current. Then, 5% skimmed milk (G5002, Servicebio, Wuhan, China) was used to seal the membranes for 1.5 h. After removing the milk, the membranes were washed 3 times with TBST. The PVDF membranes were incubated with the primary antibody overnight, and β-actin (8H10D10, cell signal technology, Danvers, MA, USA) was used as an internal control. The membranes were then washed three times with TBST and incubated with rabbit polyclonal secondary antibody for 1 h. The membrane was exposed after adding a developer to the membrane.

### 4.7. Statistical Analysis

All of the experiments were performed in triplicates, and the data have been presented as the mean ± standard deviation. One-way ANOVA or Student’s *t*-test using SPSS 18.0 (SPSS, Chicago, IL, USA) was conducted. *p* < 0.05 was considered statistically significant.

## 5. Conclusions

In this study, we isolated samwinol from *D. heterophyllum* for the first time and confirmed that samwinol could prevent the neuroinflammation induced by Aβ_25–35_ in PC-12 cells by modulating the activation of the ERK pathway. The involved mode of action was associated with the activation of the Nrf2/HO-1 pathway, which was attributed to the suppression of oxidative stress. In the future, additional mechanistic studies will be carried out to provide complete evidence for the potential of samwinol in the treatment of AD.

## Figures and Tables

**Table 1:** The binding energy and ligand interactions between samwinol and Nrf2, HO-1 as well as NQO1.

Proteins	Binding Energy (Kcal/mol)	Ligand Interactions
Nrf2	−8.40	VAL512 (Conventional Hydrogen Bond), ILE559 (Conventional Hydrogen Bond, Pi-Sigma), VAL608 (Conventional Hydrogen Bond), VAL606 (Carbon Hydrogen Bond, Alkyl), VAL463 (Carbon Hydrogen Bond), ALA510 (Carbon Hydrogen Bond), VAL418 (Carbon Hydrogen Bond), CYS513 (Alkyl, Pi-Alkyl)
HO-1	−7.23	ARG183 (Conventional Hydrogen Bond), LEU138(Pi-Sigma, Pi-Pi Stacked), GLY139 (Carbon Hydrogen Bond), HIS25 (Conventional Hydrogen Bond, Pi-Alkyl), PHE207 (Pi-Pi Stacked, Amide-Pi Stacked), ARG136 (Conventional Hydrogen Bond), ASN210 (Conventional Hydrogen Bond), TYR134 (Pi-Alkyl)
NQO1	−5.33	GLU185 (Conventional Hydrogen Bond), LYS270 (Conventional Hydrogen Bond), ILE269 (Carbon Hydrogen Bond), ARG210 (Alkyl, Pi-Alkyl), LYS209 (Alkyl, Pi-Alkyl)

## References

[1] Zheng W., Wang Q., Lu X., Shi Q., Zou J., Tao Y., Wang P. (2016). Protective Effects of Dracocephalum heterophyllum in ConA-Induced Acute Hepatitis. Mediat. Inflamm..

[2] Shi Q.Q., Dang J., Wen H.X., Yuan X., Tao Y.D., Wang Q.L. (2016). Anti-hepatitis, antioxidant activities and bioactive compounds of Dracocephalum heterophyllum extracts. Bot. Stud..

[3] Zhang C., Li H., Yun T., Fu Y., Liu C., Gong B., Neng B. (2008). Chemical composition, antimicrobial and antioxidant activities of the essential oil of Tibetan herbal medicine Dracocephalum heterophyllum Benth. Nat. Prod. Res..

[4] Chander R., Maurya A.K., Kumar K., Kumari S., Kumar R., Agnihotri V.K. (2022). In vitro antidiabetic and antimicrobial activity of Dracocephalum heterophyllum Benth. essential oil from different sites of North-western Himalayas India. Nat. Prod. Res..

[5] Bian J., Wang K., Wang Q., Wang P., Wang T., Shi W., Ruan Q. (2020). Dracocephalum heterophyllum (DH) Exhibits Potent Anti-Proliferative Effects on Autoreactive CD4(+) T Cells and Ameliorates the Development of Experimental Autoimmune Uveitis. Front. Immunol..

[6] Lu Y., Wu N., Fang Y., Shaheen N., Wei Y. (2017). An automatic on-line 2,2-diphenyl-1-picrylhydrazyl-high performance liquid chromatography method for high-throughput screening of antioxidants from natural products. J. Chromatogr. A.

[7] Lv Y., Wang Z., Wu Q., Fang Y., Wang Q., Li G., Dang J. (2022). Preparation and Antioxidant Activities of Phenylethanoids from Dracocephalum heterophyllum. Separations.

[8] Dang J., Chen C., Ma J., Dawa Y., Wang Q., Tao Y., Wang Q., Ji T. (2020). Preparative isolation of highly polar free radical inhibitor from Floccularia luteovirens using hydrophilic interaction chromatography directed by on-line HPLC-DPPH assay. J. Chromatogr. B Anal. Technol. Biomed. Life Sci..

[9] Feng J., Xiao Y., Guo Z., Yu D., Jin Y., Liang X. (2011). Purification of compounds from Lignum Dalbergia Odorifera using two-dimensional preparative chromatography with Click oligo (ethylene glycol) and C18 column. J. Sep. Sci..

[10] Querfurth H.W., LaFerla F.M. (2010). Alzheimer’s disease. N. Engl. J. Med..

[11] Tarawneh R., Holtzman D.M. (2012). The clinical problem of symptomatic Alzheimer disease and mild cognitive impairment. Cold Spring Harb. Perspect. Med..

[12] Terry R.D., Masliah E., Salmon D.P., Butters N., DeTeresa R., Hill R., Hansen L.A., Katzman R. (1991). Physical basis of cognitive alterations in Alzheimer’s disease: Synapse loss is the major correlate of cognitive impairment. Ann. Neurol..

[13] Katsumoto A., Takeuchi H., Takahashi K., Tanaka F. (2018). Microglia in Alzheimer’s Disease: Risk Factors and Inflammation. Front. Neurol..

[14] Tönnies E., Trushina E. (2017). Oxidative Stress, Synaptic Dysfunction, and Alzheimer’s Disease. J. Alzheimers Dis..

[15] Ahmad S.I., Ali G., Muhammad T., Ullah R., Umar M.N., Hashmi A.N. (2020). Synthetic β-hydroxy ketone derivative inhibits cholinesterases, rescues oxidative stress and ameliorates cognitive deficits in 5XFAD mice model of AD. Mol. Biol. Rep..

[16] Hamley I.W. (2012). The amyloid beta peptide: A chemist’s perspective. Role in Alzheimer’s and fibrillization. Chem. Rev..

[17] Zhang M., Qian C., Zheng Z.G., Qian F., Wang Y., Thu P.M., Zhang X., Zhou Y., Tu L., Liu Q. (2018). Jujuboside A promotes Aβ clearance and ameliorates cognitive deficiency in Alzheimer’s disease through activating Axl/HSP90/PPARγ pathway. Theranostics.

[18] Masters C.L., Simms G., Weinman N.A., Multhaup G., McDonald B.L., Beyreuther K. (1985). Amyloid plaque core protein in Alzheimer disease and Down syndrome. Proc. Natl. Acad. Sci. USA.

[19] Mawuenyega K.G., Sigurdson W., Ovod V., Munsell L., Kasten T., Morris J.C., Yarasheski K.E., Bateman R.J. (2010). Decreased clearance of CNS beta-amyloid in Alzheimer’s disease. Science.

[20] Mandrekar S., Jiang Q., Lee C.Y., Koenigsknecht-Talboo J., Holtzman D.M., Landreth G.E. (2009). Microglia mediate the clearance of soluble Abeta through fluid phase macropinocytosis. J. Neurosci..

[21] Griffin W.S. (2013). Neuroinflammatory cytokine signaling and Alzheimer’s disease. N. Engl. J. Med..

[22] Sun X.Y., Li L.J., Dong Q.X., Zhu J., Huang Y.R., Hou S.J., Yu X.L., Liu R.T. (2021). Rutin prevents tau pathology and neuroinflammation in a mouse model of Alzheimer’s disease. J. Neuroinflamm..

[23] Hou Y., Wei Y., Lautrup S., Yang B., Wang Y., Cordonnier S., Mattson M.P., Croteau D.L., Bohr V.A. (2021). NAD(+) supplementation reduces neuroinflammation and cell senescence in a transgenic mouse model of Alzheimer’s disease via cGAS-STING. Proc. Natl. Acad. Sci. USA.

[24] Tang L., Xiang Q., Xiang J., Zhang Y., Li J. (2021). Tripterygium glycoside ameliorates neuroinflammation in a mouse model of Aβ25-35-induced Alzheimer’s disease by inhibiting the phosphorylation of IκBα and p38. Bioengineered.

[25] Xiao H.H., Dai Y., Wong M.S., Yao X.S. (2014). New lignans from the bioactive fraction of Sambucus williamsii Hance and proliferation activities on osteoblastic-like UMR106 cells. Fitoterapia.

[26] Bandoniene D., Murkovic M. (2002). The detection of radical scavenging compounds in crude extract of borage (*Borago officinalis* L.) by using an on-line HPLC-DPPH method. J. Biochem. Biophys. Methods.

[27] Zhang Y.P., Shi S.Y., Xiong X., Chen X.Q., Peng M.J. (2012). Comparative evaluation of three methods based on high-performance liquid chromatography analysis combined with a 2,2’-diphenyl-1-picrylhydrazyl assay for the rapid screening of antioxidants from Pueraria lobata flowers. Anal. Bioanal. Chem..

[28] Wang W., Jiao L., Tao Y., Shao Y., Wang Q., Yu R., Mei L., Dang J. (2019). On-line HPLC-DPPH bioactivity-guided assay for isolated of antioxidative phenylpropanoids from Qinghai-Tibet Plateau medicinal plant Lancea tibetica. J. Chromatogr. B Anal. Technol. Biomed. Life Sci..

[29] Bedard K., Krause K.H. (2007). The NOX family of ROS-generating NADPH oxidases: Physiology and pathophysiology. Physiol. Rev..

[30] Dröge W. (2002). Free radicals in the physiological control of cell function. Physiol. Rev..

[31] Mittal M., Siddiqui M.R., Tran K., Reddy S.P., Malik A.B. (2014). Reactive oxygen species in inflammation and tissue injury. Antioxid. Redox Signal..

[32] Liang D., Li F., Fu Y., Cao Y., Song X., Wang T., Wang W., Guo M., Zhou E., Li D. (2014). Thymol inhibits LPS-stimulated inflammatory response via down-regulation of NF-κB and MAPK signaling pathways in mouse mammary epithelial cells. Inflammation.

[33] Song Z.M., Liu F., Chen Y.M., Liu Y.J., Wang X.D., Du S.Y. (2019). CTGF-mediated ERK signaling pathway influences the inflammatory factors and intestinal flora in ulcerative colitis. Biomed. Pharmacother..

[34] Bellezza I., Giambanco I., Minelli A., Donato R. (2018). Nrf2-Keap1 signaling in oxidative and reductive stress. Biochim. Biophys. Acta Mol. Cell Res..

[35] Hennig P., Garstkiewicz M., Grossi S., Di Filippo M., French L.E., Beer H.D. (2018). The Crosstalk between Nrf2 and Inflammasomes. Int. J. Mol. Sci..

[36] Pan X., Kadla J.F., Ehara K., Gilkes N., Saddler J.N. (2006). Organosolv ethanol lignin from hybrid poplar as a radical scavenger: Relationship between lignin structure, extraction conditions, and antioxidant activity. J. Agric. Food Chem..

[37] Wang L., Wang S., Yang S., Guo X., Lou H., Ren D. (2012). Phenolic alkaloids from the aerial parts of Dracocephalum heterophyllum. Phytochemistry.

[38] Youn K., Jun M. (2020). Geraniin Protects PC12 Cells Against Aβ(25-35)-Mediated Neuronal Damage: Involvement of NF-κB and MAPK Signaling Pathways. J. Med. Food.

[39] Forrester S.J., Kikuchi D.S., Hernandes M.S., Xu Q., Griendling K.K. (2018). Reactive Oxygen Species in Metabolic and Inflammatory Signaling. Circ. Res..

[40] Ding X., Jian T., Wu Y., Zuo Y., Li J., Lv H., Ma L., Ren B., Zhao L., Li W. (2019). Ellagic acid ameliorates oxidative stress and insulin resistance in high glucose-treated HepG2 cells via miR-223/keap1-Nrf2 pathway. Biomed. Pharmacother..

